# Companion Animals as Buffer against the Impact of Stress on Affect: An Experience Sampling Study

**DOI:** 10.3390/ani11082171

**Published:** 2021-07-22

**Authors:** Mayke Janssens, Erik Janssens, Jannes Eshuis, Johan Lataster, Marianne Simons, Jennifer Reijnders, Nele Jacobs

**Affiliations:** 1 Faculty of Psychology, Open University, 6401 DL Heerlen, The Netherlands; erikjanssens@casema.nl (E.J.); jannes.eshuis@ou.nl (J.E.); johan.lataster@ou.nl (J.L.); marianne.simons@ou.nl (M.S.); jennifer.reijnders@ou.nl (J.R.); nele.jacobs@ou.nl (N.J.); 2 Department of Psychiatry and Psychology, School for Mental Health and Neuroscience, Maastricht University Medical Centre, 6200 MD Maastricht, The Netherlands

**Keywords:** human–animal interaction, human–animal bond, animal companionship, pet-effect, buffering model, mental health, daily life, ecological momentary assessment, ambulatory assessment

## Abstract

**Simple Summary:**

Companion animals have been identified as a unique source of social support and as contributors to mental wellbeing. In order to identify whether a companion animal buffers against the aversive effects of stress on affect or whether it has a general effect on its owner’s affective state, this study uses a mobile app to question participant’s stress levels for five consecutive days (ten times a day in the moment), the presence and interactions with their companion animal, and on their affective states. The results show that the presence of a companion animal buffers against the detrimental effects of stress on positive affect. The association between the presence of a companion animal and positive affect is only present when experiencing stress. When not under stress, positive affect does not benefit from the presence of a companion animal. Positive affect, however, does benefit from the interaction with a companion animal: In the presence of a companion animal, individuals experience less negative affect. These effects are present in all levels of stress. In conclusion, having a companion animal around alleviates negativity, interacting with it increases positivity, and, when an individual is under stress, simply having your cat or dog around helps you to retain your positive feelings.

**Abstract:**

Companion animals have been identified as a unique source of social support and as contributors to mental wellbeing. This study uses the Experience Sampling Method to test whether this effect is due to stress-buffering. A total of 159 dog and cat owners responded to a series of randomly scheduled questionnaires on their smartphones. At each measurement moment, they reported in whether a pet is present at that moment and to what extent they have interacted with the pet. They also reported on stressful activities and events and on their current positive (PA) and negative (NA) affect. Multilevel regression analyses showed that when a companion animal was present (vs. absent) the negative association between stress and PA is less pronounced (event stress: B = 0.13, *p* = 0.002, 95% CI = 0.05; 0.21 activity stress: B = 0.08, *p* < 0.001, 95% CI = 0.04; 0.12). No additional main effect was revealed when tested in a subsample of records that reported low or no stress. Main effects were found for the presence of a companion animal on negative affect (B = 0.08, *p* < 0.001; 95% CI = 0.12; 0.05) and for interacting with a companion animal on positive affect (B = 0.06, *p* < 0.001; 95% CI = 0.04; 0.08). This shows that the presence of a companion animal buffers against the negative consequences of stress on positive affect, indicating stress-buffering as a mechanism behind the pet-effect. It is, however, not the only mechanism and more research is required to further elucidate how companion animals contribute to human wellbeing.

## 1. Introduction

Over the last few decades, a significant amount of research regarding the effect of human–animal interaction (HAI) on human wellbeing has been conducted. In the case of companion animals, this effect has since been termed the pet-effect and refers to the idea that improvements in human health, psychological wellbeing, and longevity are promoted by living or interacting with a companion animal [1].

The pet-effect has been reviewed by several authors [2,3,4], reporting positive impacts of companion animals on, for instance, mood, self-esteem, and social skills. Interacting with companion animals has additionally been linked to a reduction in stress related parameters, such as heart rate, cortisol levels, and blood pressure. Although an increasing body of studies claim to prove the beneficial effects of HAI, there are also studies reporting no effect or even adverse effects [2,3,4]. This outcome variance in HAI research can at least partly be explained by variations in design and methodology [3,4]. A variety of designs have been used to answer similar questions and studies are afflicted with methodological limitations and constraints. These limitations are related to small sample sizes and homogeneous samples; the lack of control groups or variations in type of control conditions; and lack of standardized measures or the use of measures that are not sensitive to change following human–animal interaction [3,4,5,6,7]. Moreover, the heterogeneity in which humans perceive, respond to, and interact with their companion animal may contribute to the outcome variance [6]. This heterogeneity can be due to demographic characteristics, but can also be due to human personality traits and attachment styles [7]. The present study therefore uses the Experience Sampling Method (ESM) to study the relation between stress, HAI, and wellbeing in real life. This method provides within-person evaluations of stress, HAI, and wellbeing by minimizing many methodological constraints currently present in HAI research and by maximizing ecological validity [8,9,10,11].

### 1.1. Stress and Social Support

Research into the effects of HAI has grown immensely over the last decade or two. Several aspects of wellbeing have been studied in relation to companion animals; however, stress related mental health is the outcome that is most often targeted with HAI [12]. Companion animals have shown to buffer the autonomic response to acute stress [13] and provide significant cardiovascular benefits [1,13]. Most research into the stress-buffering effect of companion animals used experimental stress-tasks by manipulating stress in a laboratory and mostly focusing on the physiological outcome measures [14]. These objective parameters, however, are not always associated with measures of subjective distress and negative affect [9] and, in real life, stress and the stress response originate in the interaction with environmental contexts that vary over time. In the present study, we therefore focus on psychological stressors in daily life such as daily hassles and small disturbances. We study the subjective appraisal of stress and the affective response to this stress and examine the role that HAI has in the association between these two.

A prior ESM study with respect to the effects of HAI in daily lives [15] revealed that the presence as well as the interaction with a companion animal is associated with different aspects of emotional wellbeing, which shows that, outside of the laboratory, companion animals also exert their influence. This study, however, only showed that a pet-effect can be detected in daily life and the impacts that companion animals have on the stress-response in the context of daily life remains unclear. This reflects a general tendency to focus on questions regarding whether and when the effects of HAI emerge as opposed to questions related to how or why HAI affects human wellbeing. There are, however, several promising mechanisms to be considered in attempting to clarify why companion animals might be able to improve human health. The psychological mechanisms attempting to explain the effects of HAI on mental wellbeing focus on the social aspects of the human-companion animal bond. Companion animals can serve as social lubricants or social catalysts and thus impacts human wellbeing by stimulating positive social interactions and social support. They have, however, also been shown to function as a source of social support themselves [1,5,6,16,17,18]. The activation of the oxytocin system by the companion animal has been presented as an important neurobiological mechanism underlying the positive effect of HAI [2,5,6,17,19]. Oxytocin plays an important role in attachment and social behavior and has a stress-regulating effect. The interactions with companion animals trigger the release of oxytocin and the closer the relation between the human and the accompanying animal, the more oxytocin is released [17,19]. Although oxytocin and social support are discussed as a different mechanism by some researchers [5,17], others [6] place social support and oxytocin under the same mechanism. They pose that both mechanisms mediate through the same neurological system, which is the HPA axis, with the result of activating the dopamine reward system, which inhibits stress responses.

### 1.2. Buffering Model or Main Effect Model

Social support has been identified as a protective factor in the adverse effects of stress. Different types of social support, provided for instance by friends and family, tend to reduce the level of stress in an individual [20]. As previously mentioned, research has determined that companion animals are also providers of social support [5,6,12,16,17]. According to Cohen and Wills [21], however, the effects of social support on wellbeing are not necessarily related to stress-buffering. They postulated two pathways or models describing how social support derived from humans has a beneficial effect on wellbeing. These two models are known as the main effect model and the buffering model [21]. The buffering model proposes that social support protects (“buffers”) against the aversive effects of stress. Thus, support is only (or primarily) related to wellbeing when under stress. The main effect model attributes the positive association between social support and wellbeing to an overall beneficial effect of social support. This model proposes that the beneficial effect of social support is independent of stress. These two models are not mutually exclusive. Social support can have general beneficial effects resulting in higher wellbeing and, additionally, induces buffering effects by protecting wellbeing against the negative consequences of stressful experiences.

Taken together, it is known that companion animals function as a source of social support [5,6,12,16,17], are linked to stress-reduction [12,13,16], and can have a positive impact on wellbeing [1,3,4]. However, it has not been established through which pathway HAI exerts its effect. Further investigation into the role that HAI plays in the association between stress and affect might elucidate the mechanisms behind the pet-effect and shed light on inconsistencies in previous research.

### 1.3. The Present Study

The present study, therefore, adopts experience sampling method [9,10,11] by using repeated (random) sampling of momentary behaviors and experiences over the course of time to gather data in the natural environment of participants and their companion animals. This method allows for in-the-moment assessment of the subjective appraisal of stressors and daily life hassles as they appear in the natural flow of daily life. At the same moment, data with respect to positive and negative affective states (indicating wellbeing) as well as the presence and interaction with the companion animal are gathered, providing a unique view on the relationship between the human and their companion animal in various states of stress. 

The aim of the present study is to investigate whether HAI is related to wellbeing only when experiencing stress (the buffering model) or whether this effect (also) exists irrespective of stress (the main effect model). This main effect has already been shown in a prior study with a smaller sample [15], but stress was not included in that analysis. The present study extends on that study in order to test for the role of stress in the pet-effect. We will first establish if there is an association between stress and affect and whether HAI moderates this effect (the buffering model). Depending on the results, we will then conduct one of two investigations: (1) If support for the buffering model is found, we will investigate whether an additional main effect irrespective of stress is present (the combined model) by testing the association between HAI and affect in the absence of stress. If (2) support for the buffering model is not found, we will investigate whether a true main effect is present by testing the association between HAI and affect in the entire sample.

## 2. Materials and Methods

### 2.1. Participants

Participants have been recruited by both undergraduate and graduate students of the Open University (Open Universiteit, the Netherlands). Participants were recruited in the students’ own environment and through their local veterinarian or pet shops. Participants were required to (i) have reached the age of 18; (ii) live with at least one dog and/or cat; (iii) have access to a smartphone for the duration of the survey period; (iv) have a sufficient command of the Dutch language to assure that they understood instructions and could provide informed consent. Participation in the study was voluntary and all participants provided (digital) informed consent. The study was approved by the research ethics committee of the Open University (U2016/00165/CBO).

### 2.2. Procedure

In order to collect demographic characteristics and information concerning their companion animal, participants were first asked to fill out an online questionnaire. After completion, participants were requested to install the RealLife Exp app [22] on their smartphones. This application served to collect momentary data of the participants. For five consecutive days, participants received ten notifications on their smartphones each day that were randomly scheduled between 7:30 a.m. and 22:30 p.m. At each notification, participants were questioned about their current affect, activities, location, social contacts, and events as well as the presence of and interaction with their companion animal (s). Participants were instructed to respond immediately upon the notification. In order to avoid memory distortion and to optimize reliability, the ESM questionnaire expired after 15 min and was no longer available to the participants. Participants were instructed to (self)select a sampling period that included a mixture of work/study related days and (at least) two days with no work or school related activities since (for most people) the presence of an animal is not an option during work or school related activities. Furthermore, the survey period was required to reflect a normal week and was not to be scheduled when extraordinary (life) events were planned (e.g., a marriage or a holiday). For the sake of the reliability of data, in the case where a participant submitted less than 33% out of the 50 valid reports, all data of this participant were excluded from analysis [23]. Part of the sample (*N* = 55) used in this study was also used in the study by Janssens et al. [15]. In order to increase power to perform more complex analyses, the sample was extended under the same protocol. 

### 2.3. Measures

#### 2.3.1. Stress

Momentary stress was conceptualized as the subjectively appraised stressfulness of distinct events and activities [9,15]. Activity Stress was assessed using the following questions about their current activity: “I would rather do something else”, “this takes effort”, and “I am good at this”. All answers were measured on a 7-point Likert scale (1 = not at all, 7 = very). The third item was reverse-coded and the mean score of these items indicate the level of stress. A low score represents a low level of stress and a high score represents a high level of stress. Event Stress was measured by asking participants to rate the most important event since the previous notification on a bipolar Likert scale uses the question, “This event was… (−3 = very unpleasant, +3 = very pleasant).” Values indicating pleasurable events (+1 to +3) were recoded to 0 and responses were reverse-coded to allow high scores to reflect high levels of stress (0 = no stress and 3 = high stress). 

#### 2.3.2. HAI

*Pet Presence* indicates whether or not a companion animal was present, using the question “at this moment my pet is present” (0 = no, 1 = yes). Pet Interaction indicates the level of interaction with a companion animal. When a companion animal was present, the follow-up question “We are interacting” was asked. Participants were requested to rate the interaction on a 7-point Likert scale (1 = not at all, 7 = very) [15]. 

#### 2.3.3. Affect

In accordance with previous ESM studies, participants’ affective states were assessed using mood related adjectives derived from the Positive And Negative Affect Schedule (PANAS [24]). The scale was composed of items that showed high loadings on both positive and negative affect latent factors and sufficient within-person variability in previous ESM studies [15,25,26,27]. The items of this scale covered a broad range of affect across the dimensions of “valence” (positive–negative) and of “arousal” (high–low) [28]. The Positive Affect (PA) scale comprised of the statements such as “I feel cheerful”, “I feel satisfied”, “I feel happy”, and “I feel enthusiastic”, which were rated on a 7-point Likert scale (1 = not at all, 7 = very). The mean score of these items indicated the level of PA with a high score reflecting more positive affect. The Negative Affect (NA) scale consisted of the statements such as “I feel insecure”, “I feel lonely”, “I feel anxious”, “I feel irritated”, “I feel sad”, and “I feel guilty”, which were all rated on a 7-point Likert scale (1 = not at all, 7 = very). The mean score of these items indicated the level of NA with a higher score reflecting more negative affect.

### 2.4. Analyses

ESM data have a hierarchical (multilevel) structure: Multiple momentary assessment points (level 1) are nested within subjects (level 2). In order to take this multilevel structure into account, multilevel regression modeling using the lme function of nlme [29] was performed in RStudio. To do justice to the within person effect of the individual participants, stress measures were centered around the individual’s mean. 

In order to test for an association between stress and affect, multilevel regression analyses were performed by entering, respectively, NA and PA as dependent variables and Activity Stress and Event Stress as independent variables. In order to test for the buffering model, HAI and the interaction values of HAI and stress (HAI *stress) were added to the model. This was performed separately for the two HAI measures (Pet Presence and Pet Interaction), the two stress measures (Activity Stress and Event Stress), and the two affect measures (PA and NA).

Continuation of the analyses was dependent on whether or not a buffering model was found. When no interaction effect was found (i.e., no indication for a buffering model), the analysis continued with testing for a main effect: The association between Pet Presence and/or the association between Pet Interaction and, respectively, NA or PA was tested in the complete sample. Multilevel regression models were tested entering the affect measures as dependent variables and either Pet Presence or Pet Interaction as the independent variable.

When an interaction effect was found (providing evidence for the buffering model), an additional main effect (irrespective of stress) was tested. Using multilevel regression models, the association between Pet Presence and/or Pet Interaction and NA or PA was tested in a subsample of observations for which low or no levels of stress were reported (Activity Stress < 2, Event Stress = 0).

In order to reduce the probability of type I error due to the number of models that were tested, family-wise error-corrected *p* values (*p*FWE) were computed. For each type of model (family), unadjusted *p* values were multiplied by the number of tests in that family (*N*). Families used to compute corrected *p* values were the association between stress and affect (*N* = 4), the interaction models (*N* = 8), and the follow-up analyses to test for a main effect (*N* = 5). 

Age, gender, and the presence of other people (“are you alone”, yes/no) were considered as possible confounders in the analyses and were included as covariates. All models accounted for serial dependency allowing residuals to be correlated over time (satisfying AR (1) model) and allowed for intercepts and slopes to vary randomly across individuals.

## 3. Results

### 3.1. Descriptive Statistics and Reliability Analysis

A total of 223 participants have participated in this study. The records of 64 participants were excluded from the analyses based on insufficient valid ESM reports. The sample used for analysis was based on the responses of 159 participants. In total, the participants responded 4872 times to the questions asked in the RealLife Exp app. In 59.9% (2.920 records) of these responses, their companion animals were present. For details of the sample, see Table 1.

A reliability analysis was carried out on the affect scales. Comprising six items, Cronbach’s alpha of the NA scale showed α _(aggregated)_ = 0.83; on centered items, Cronbach’s alpha showed α _(within)_ = 0.67. The PA scale, which comprises four items, showed Cronbach’s alpha _(aggregated)_ = 0.89; Cronbach’s alpha _(within)_ = 0.84. Full details of descriptive data are presented in Table 1.

### 3.2. Association between Stress and Affect

Multilevel random regression analyses confirmed the positive association between both stress-measures and NA, as well as the negative association between both stress-measures and PA. When higher levels of stress were reported, respondents indicated that they experienced more negative and less positive affects. The results are presented in Table 2. 

### 3.3. Pet Presence as Buffer for the Effects of Stress on Affect

A significant buffering effect was found for the interaction between stress and Pet Presence in the model of PA. The effect of the interaction between Event Stress and Pet Presence was B = 0.13 (*p* = 0.002, CI = 0.05; 0.21) and, for the interaction between Activity Stress and Pet Presence, the effect was B = 0.08 (*p* < 0.001, CI = 0.04; 0.12). See Table 3 for full details of the analyses. 

### 3.4. Main Effect of Pet Presence and Pet Interaction 

As no interaction effects of Stress and Pet Presence were observed in the model of NA and no interaction effects of Stress and Pet Interaction were found in either of the Affect models, the main effects can be interpreted for the association between Pet Interaction and PA and NA and for the association between Pet Presence and NA. This was performed in order to assess evidence for the main effect model, stating that HAI is directly related to aspects of wellbeing. Main effects were found for the association between the presence of a companion animal and NA (B = −0.08, *p* < 0.001; CI = −0.12; −0.05) and the interaction with a companion animal and PA (B = 0.06, *p* < 0.001; CI = 0.04; 0.08). Thus, the presence of a companion animal has a general diminishing effect on its owner’s negative affect, while interacting with a companion animal elevates positive affect in the owner. The results are presented in Table 4.

### 3.5. Combined Model and Association between Pet Presence and PA in Absence of Stress

Since the interaction effects of both stress measures and Pet Presence in the model of PA were significant, the main effects of Pet Presence on PA were tested in a subsample of records that registered low levels of stress. This was performed to test whether the presence of a companion animal induces a main effect in addition to buffering against the impact of stress on PA. For Event Stress, the low stress subsample (Event Stress = 0) comprised 4346 responses of 159 participants. For Activity Stress, the low stress subsample (Activity Stress ≤ 2) comprised 2390 responses of 154 participants. In both of these subsamples, the *p* value (corrected and uncorrected) failed to reach significance; no additional main effect was revealed. See the results in Table 5.

## 4. Discussion

This study was designed to examine whether HAI in daily life buffers against the impact of stress on affect or whether HAI has a more general beneficial effect on wellbeing that is consistent with the buffer model or the main effect model of social support [21]. A buffering effect was found for the presence of a companion animal on PA; when a companion animal was present, the negative association between stressful events or activities is less pronounced than when the companion animal was not present. This stress-buffering effect of companion animals does not occur for NA and is specific for the presence of (as opposed to interacting with) a companion animal. It is a pure buffering effect; when tested in a subsample of records where no stress was reported, no additional main effect on PA was revealed. Thus, while the presence of a companion animal buffers against the negative consequences of stress on PA, pet presence does not affect PA in the absence of stress. 

The models in which HAI did not buffer the negative consequences of stress, i.e., models predicting NA (both pet presence and pet interaction) and PA (pet interaction only), were tested for a main effect of HAI. Results show that the presence (vs. interaction) of a companion animal is associated with less NA, while the interaction (vs. no interaction) with a companion animal is associated with more PA. When a companion animal is present, individuals experience less negative affect; more positive affect, however, is only experienced when the interaction with the animal is more intensive. 

### 4.1. Companion Animals as Stress-Buffer

The results show that the presence of a companion animal effectively protects against the negative consequences of stress on the positive affect. The response to a stressful situation will be less negative in the case where a companion animal is present. However, in situations where stress is limited, the presence of a companion animal seems to have no impact on the positive affective state. This indicates that beneficial effects of social support of a companion animal on positive affect are effectuated through the process of stress buffering, implying that stress is a key condition of the pet-effect.

Stress being a key condition does endorse that a large number of positive effects of HAI are linked to stress-related parameters. This explicates, for instance, the evidence found when a companion animal proved to be a successful addition in the treatment of hypertensive stockbrokers [30]. The high-stress occupational circumstances of these individuals made them a highly suitable sample to catch the stress reducing effect of a companion animal. However, less severe conditions of stress support the effect as revealed in the present study. This would, for example, clarify why a dog had no effect on the anxiety level of young adults when reading quietly but did have an effect when reading out loud [31]. Only the second condition involved stress; hence, the pet-effect enters into force. By using populations with high stress levels or the inclusion of conditions that compare stress to no-stress, an effect of HAI is more likely to be discovered. However, in studies that did not include stress as a potential variable, the effect of a companion animal might have been missed or misinterpreted. The designation of stress as a key condition of the pet-effect implies that that stress should play a central role in future studies to the effects of HAI.

The buffering effect of companion animals was, however, only found in a specific context; the presence of a companion animal (and not the interaction with a companion animal) buffered against the negative impact on positive affect (but not negative affect). The level of interaction with a companion animal, however, was only questioned when a companion animal was present. Therefore, the results do not indicate that there is no buffering-effect for the interaction with a companion animal, but that there is no additive effect over the presence of a companion animal. The presence of a companion animal buffers against the detrimental effects of stress and the intensity of the interaction seems to be irrelevant in this effect.

Our results concerning the buffering effect of companion animals are in line with the results found for social support received from human companions by Cohen and Wills [21]. They showed that (functional) social support triggered a buffering model. Interestingly, our results show that, for companion animals, this buffering effect is present for PA but not for NA. A discrepancy between the effect of HAI on PA versus NA has been shown in prior research [15] and was hypothesized to be linked to the nature and reciprocity of the interaction. A higher level of interaction is most likely characterized by a need or wish to engage with the animal in a two-way interaction comparable to social interaction between humans. Social interaction between humans has been shown to correlate differentially with PA and NA and observed to affect PA but not NA [32,33]. The presence of a companion animal, on the other hand, does not necessarily have this reciprocal nature and can be instigated by the owner (seeking proximity to the animal), by the companion animal (seeking proximity to the owner), or be a result of chance (simply happening to be in the same room). An alternative explanation, however, could be the skewed distribution of NA in our sample, resulting in low scores with relatively little variation. Not finding a buffering effect for NA could therefore also be due to a floor effect. 

### 4.2. Main Effect of a Companion Animal

The presence of a companion animal was only found to buffer against the effects of stress on PA; no stress-buffering effect was found for the interaction with a companion animal (in both affect models) or for the presence of a companion animal in the model of NA. For these three models, the main effect of HAI was therefore tested in the entire sample since stress does not influence the associations. The results revealed a differential effect for pet presence and pet interaction. The presence of (but not the interaction with) a companion is negatively associated with negative affect and a higher level of interaction with (but not the presence of) a companion is associated with higher positive affect. Thus, individuals experience less negative affect in the presence of a companion animal, but when interacting with a companion animal, the positive affective state is relatively higher. This is in line with prior research [15] that tested the effect of the presence of a companion animal and the interaction with a companion animal on positive and negative affect. In this prior study, post-hoc analyses showed a discrepancy between the passive presence of a companion animal and the (active) interaction with a companion animal. This strengthens the conclusion that the pet-effect can be found in daily life but is not an ubiquitous effect. The different aspects of the human–animal relationship seem to influence different aspects of emotional wellbeing. Although the present study did not perform post-hoc analyses to optimally differentiate between passive presence and (active) interaction, a discrepancy between aspects of the relationship between HAI, stress, and affect was found showing that the mechanisms behind the pet-effect also seem to be equivocal. In the association between pet presence and positive affect, stress is a key condition while, for the other models, stress does not seem to be a factor.

Cohen and Wills [21] also found a main effect of social support, but only for the structure of the social network. The structure of the social network is however a more stable construct not suited to be measured using ESM and the social network is broader than just the companion animal. In addition, we did not measure social support directly. Taken together, the mechanism behind the main effects of companion animals remains somewhat elusive. The effect of the presence of a companion animal and interaction with a companion animal on, respectively, NA and PA could be related to social support received from the companion animal, which shows that the support from a companion animal had a buffering effect as well as a main effect depending on the aspects of the HAI and wellbeing that are being measured. An alternative explanation is that the mechanism for the main effect is different and latent variables, such as attachment to the companion animal or specific activities with the animal, might explain the effects found.

### 4.3. Strengths and Limitations

The main strengths of this study are related to the momentary data collection. Affective states are documented in real time and minimizes retrospective bias, which is especially important when measuring affective components of wellbeing [34]. The ESM also enabled us to measure daily hassles and small disturbances from natural sources of stress [9,11]. The implicit nature of this approach allowed us to study the relationship between companion animals, stress, and affect as accurately as possible, avoiding cognitive interpretations and social desirableness. Additionally, repeated assessments over time allow each individual to be their own control condition [8,9,10,11,15]. This eliminates the impact of preexisting differences between pet owners and non-owners and constitutes an ideal control condition.

There are, however, some limitations that should be considered when interpreting the results. First, the demanding nature of this method of data collection as well as the requirement to be in the possession of a smartphone possibly resulted in a selection bias. Second, the current sample displays an overrepresentation of female participants, married participants or those that are living with a significant other, and of participants that completed higher levels of education. This limits the generalizability of the conclusions. Third, the large number of tests performed enabled us to correct for multiple testing. The issue of what constitutes multiplicity and how best to correct for this is not an easy one [35]. We decided to compute family-wise corrected *p* values based on three sets (“families”) of tests. A more strict correction, however, would not results in a different interpretation of the results. A more liberal interpretation would result in an interpretation that shows a slightly larger role for stress in the pet-effect (see Table 2, Table 3, Table 4 and Table 5 for uncorrected *p* values). Fourth, social support from a companion animal was not measured directly but by using the presence of and interaction with the companion animal as proxy. We have employed the evidence that companion animals provide social support [1,5,6,16,17,18], but the level or type of social support was not assessed. Finally, although the present study focuses on within person associations, the stress-levels in our sample were relatively low when investigating the degree to which positive affect varies within a subject in relation to the experienced stressfulness of particular events or activities. Still, a buffering effect of the companion animal was found, showing that the presence of a companion animal buffers against the negative impact of minor stresses. Whether this is also the case for higher levels of stress remains unclear. More research is needed to investigate whether this buffering effect only applies to minor stresses and hassles or also for daily life experiences that induce higher levels of stress. It is, however, important to note that these minor hassles and stresses are experienced on a daily basis and often even several times during the day. Cumulatively, the effect of HAI on the consequences of these stressful experiences can have a large impact.

## 5. Conclusions

In summary, this study shows that the presence of a companion animal buffers against the detrimental effects of stress on positive affect. The association between the presence of a companion animal and positive affect is only present when experiencing stress. When not under stress, positive affect does not benefit from the presence of a companion animal. Positive affect, however, does benefit from the interaction with a companion animal and, when in the presence of a companion animal, individuals experience less negative affect. These effects are present in all levels of stress. In conclusion, having a companion animal around alleviates negativity, interacting with it increases positivity, and, when you arere under stress, simply having your cat or dog around helps you to retain your positive feelings.

This shows again that the pet-effect is not an equivocal effect and the same holds for the mechanism behind this effect. We found clear evidence that stress is indeed a key condition of the pet-effect, indicating stress-buffering as a mechanism behind the pet-effect. The quest, however, does not end here, as under some circumstances a main effect of HAI was also found. What exactly constitutes or drives this effect remains unclear. It has been postulated that many different mechanisms or processes are involved in the positive effect of companion animals [12]. Showing a specific stress-buffering effect as well as separate main effects supports this notion. More research is needed to further elucidate the mechanisms behind the pet-effect and to clarify the contexts and elements of HAI and their impact on specific aspects of wellbeing. This will not only further the scientific basis of the pet-effect but can also have practical implications in providing insight into the specific elements of the interaction with an animal and into the mechanisms behind the positive effects of the animal that can be invoked in animal assisted interventions.

## Figures and Tables

**Table 1:** Descriptive data of sample ^a^.

Variable	Range	*M* ^b^	*SD* ^c^	Frequency	Percent
**Age**	19–71	44.2	12.8	-	-
**Gender**	-	-	-	-	-
Female	-	-	-	110	69.2
Male	-	-	-	49	30.8
Owners of dogs	-	-	-	95	59.7
Number of dogs	1–3	-	-	107	-
Owners of cats	-	-	-	86	52.2
Number of cats	1–5	-	-	138	-
**Civil status**	-	-	-	-	-
Single	-	-	-	20	12.6
Relation, not living together		-	-	15	9.4
Married/living with significant other		-	-	115	72.3
Divorced	-	-	-	8	5.0
Widowed	-	-	-	1	0.6
**Education**	-	-	-	-	-
Primary education	-	-	-	1	0.6
Lower vocational education	-	-	-	2	1.9
Intermediate secondary education	-	-	-	11	6.9
Higher secondary education	-	-	-	12	7.5
Pre-university education	-	-	-	8	5.0
Intermediate vocational education	-	-	-	34	21.4
Higher vocational education	-	-	-	62	39.0
University	-	-	-	29	18.2
**Measures**	-	-	-	-	-
Activity Stress ^d^	1.06–4.48	2.48	0.69	-	-
Event Stress ^d^	0.00–1.12	0.20	0.21	-	-
Positive Affect ^d^	2.01–6.79	4.87	0.86	-	-
Negative Affect ^d^	1.00–4.25	1.48	0.57	-	-
Companion Animal Present	-	-	-	2.920	59.9
Companion Animal Not Present	-	-	-	1.952	40.1
Pet Interaction ^d^	1.00–6.00	1.48	1.10	-	-

^a^  *N* = 158, ^b^  *M* = mean, ^c^  *SD* = standard deviation, ^d^ within subject calculations.

**Table 2:** Association between stress and affect.

-	B ^a^ (95% CI ^b^)	*p*	*p*FWE ^c^
NA	*-*	-	-
Event Stress	0.19 (0.17; 0.21)	<0.001	<0.001
Activity Stress	0.16 (0.14; 0.17)	<0.001	<0.001
PA	-	-	-
Event Stress	−0.41 (−0.45; −0.37)	<0.001	<0.001
Activity Stress	−0.40 (−0.42; −0.38)	<0.001	<0.001

^a^ B = unadjusted Beta, ^b^ CI = confidence interval, ^c^ Family-wise error-corrected *p* values (*p*FWE) based on 4 tests (*N* = 4).

**Table 3:** Interaction between companion animal (presence and interaction) and stress (activity related and event related) in the model of NA and PA (buffering model).

-	B ^a^ (95% CI ^b^*)*	*p*	*p*FWE ^c^
NA	*-*	*-*	-
Event Stress * Pet Presence	−0.05 (−0.09; 0.00)	0.041	0.331
Activity Stress * Pet Presence	0.01 (−0.02; 0.03)	0.619	>0.999
Event Stress * Pet Interaction	−0.01 (−0.03; 0.00)	0.174	>0.999
Activity Stress * Pet Interaction	0.00 (−0.00; 0.01)	0.248	>0.999
PA	-	-	-
Event Stress * Pet Presence	0.13 (0.05; 0.21)	0.002	0.012
Activity Stress * Pet Presence	0.08 (0.04; 0.12)	<0.001	0.002
Event Stress * Pet Interaction	0.00 (−0.03; 0.02)	0.728	>0.999
Activity Stress * Pet Interaction	−0.01 (−0.03; 0.00)	0.034	0.268

Note: ^a^ B = unadjusted beta, ^b^ CI = confidence interval, ^c^ Family-wise error-corrected *p* values (*p*FWE) based on 8 tests (*N* = 8); * indicates the interaction term.

**Table 4:** Main effect: effect of pet presence and pet interaction.

-	B (95% CI)	*p*	*p*FWE
NA	-	-	-
Pet Presence	−0.08 (−0.12; −0.05)	<0.001	<0.001
Pet Interaction	−0.01 (−0.02; −0.00)	0.012	0.062
PA	-	-	-
Pet Interaction	0.06 (0.04; 0.08)	<0.001	<0.001

Note: Family-wise error-corrected *p* values (*p*FWE) for follow-up analyses based on 5 tests (*N* = 5).

**Table 5:** Associations between pet presence and positive affect in a subsample of records for which reported stress-levels are low (combined model).

-	-	B (95% CI)	*p*	*p*FWE
Event Stress = 0	-	0.06 (−0.01; 0.13)	0.115	0.574
Activity Stress ≤ 2	-	−0.04 (−0.12; 0.05)	0.408	>0.999

Note: Family-wise error-corrected *p* values (*p*FWE) for follow-up analyses based on 5 tests (*N* = 5).

## Data Availability

The data presented in this study are available upon request from the corresponding author.
